# The Anti-Proliferative and Apoptotic Effects of Rutaecarpine on Human Esophageal Squamous Cell Carcinoma Cell Line CE81T/VGH In Vitro and In Vivo

**DOI:** 10.3390/ijms23052843

**Published:** 2022-03-05

**Authors:** Li-Yu Wang, Shu-Lan Yeh, Shih-Tien Hsu, Chao-Hsiang Chen, Chien-Chih Chen, Cheng-Hung Chuang

**Affiliations:** 1 Department of Nutrition, Hungkuang University, Taichung 433304, Taiwan; u99c206@gmail.com; 2 Department of Food Science and Biotechnology, National Chung Hsing University, Taichung 40227, Taiwan; 3 Department of Nutrition, Chung Shan Medical University, Taichung 40201, Taiwan; suzyyeh@csmu.edu.tw; 4 Department of Obstetrics and Gynecology and Women’s Health, Taichung Veterans General Hospital, Taichung 40705, Taiwan; sthsu@vghtc.gov.tw; 5 Center for General Education, Ling Tung University, Taichung 408284, Taiwan; 6 School of Medicine, China Medical University, Taichung 404333, Taiwan; 7 Ko Da Pharmaceutical Co., Ltd., Taoyuan City 32459, Taiwan; 8 Graduate Institute of Pharmacognosy, Taipei Medical University, Taipei 110, Taiwan; 9 Department of Cosmetic Science, Chang Gung University of Science and Technology, Taoyuan City 33303, Taiwan; chen37426972@gmail.com

**Keywords:** esophageal cancer, rutaecarpine, apoptosis, p53 gene, *Evodia rutaecarpa*

## Abstract

The overall five-year survival rate for patients with esophageal cancer is low (15 to 25%) because of the poor prognosis at earlier stages. Rutaecarpine (RTP) is a bioalkaloid found in the traditional Chinese herb *Evodia rutaecarpa* and has been shown to exhibit anti-proliferative effect on tumor cells. However, the mechanisms by which RTP confer these effects and its importance in esophageal squamous cell carcinoma treatment remain unclear. Thus, in the present study, we first incubated human esophageal squamous cell carcinoma cell line, CE81T/VGH, with RTP to evaluate RTP’s effects on tumor cell growth and apoptosis. We also performed a xenograft study to confirm the in vitro findings. Furthermore, we determined the expression of p53, Bax, bcl-2, caspase-3, caspase-9, and PCNA in CE81T/VGH cells or the tumor tissues to investigate the possible mechanisms. All the effects of TRP were compared with that of cisplatin. The results showed that RTP significantly inhibits CE81T/VGH cell growth, promotes arrest of cells in the G_2_/M phase, and induces apoptosis. Consistently, the in vivo study showed that tumor size, tumor weight, and proliferating cell nuclear antigen protein expression in tumor tissue are significantly reduced in the high-dose RTP treatment group. Furthermore, the in vitro and in vivo studies showed that RTP increases the expression of p53 and Bax proteins, while inhibiting the expression of Bcl-2 in cancer cells. In addition, RTP significantly increases the expression of cleaved caspase-9 and cleaved caspase-3 proteins in tumor tissues in mice. These results suggest that RTP may trigger the apoptosis and inhibit growth in CE81T/VGH cells by the mechanisms associated with the regulation of the expression of p53, Bax, Bcl-2, as well as caspase-9 and caspase-3.

## 1. Introduction 

Esophageal cancer is characterized as a highly malignant tumor. According to the World Health Organization’s 2020 statistics, esophageal cancer is the sixth leading cause of cancer-related deaths and the seventh most common cancer worldwide [1]. There are two main histopathological subtypes of esophageal cancer: squamous cell carcinoma and adenocarcinoma, with the former being the dominant form in Asia and Africa [1,2]. Esophageal squamous cell carcinoma (ESCC) is caused primarily by smoking and the consumption of alcohol and hot beverages, while adenocarcinoma is associated with obesity, smoking, and gastroesophageal reflux disease [2] Because there are no early detection strategies for early esophageal cancer, it is largely diagnosed during its later stages [3]. Thus, the overall five-year survival rate for people with esophageal cancer is less than 20% [1]. Clinical treatments for esophageal cancer include surgery, radiotherapy, chemotherapy, and immunotherapy [4]. At present, the most common drugs used in treatment include cisplatin (*cis*-diamminedichloroplatinum (II), CDDP), 5-fluorouracil, and docetaxel [5,6]. During treatment, patients often develop resistance to the drugs or suffer side effects, shortening the five-year survival rate to a mere 15–25% [7,8]. Against this backdrop, there is a pressing need to develop effective drugs with minimal side effects for the treatment of esophageal cancer.

Purified chemical substances extracted from natural plants can offer anticancer properties by inhibiting cell growth or inducing apoptosis. In traditional Chinese medicine, the dried fruits of *Evodia rutaecarpa* and the Rutaceae family are used to treat various ailments, including headaches, dermatophytoses, gastric ulcers, and aphthae [9,10]. In recent years, scholars have isolated Elarge amounts of bioactive constituents from *Evodia rutaecarpa*, such as alkaloids, saponins, and phenols [10,11], of which rutaecarpine (RTP, an alkaloid) has been demonstrated to be the major active compounds [12]. RTP has many properties, including vasodilatory [13], antiplatelet [14], anti-inflammatory [15,16], anti-angiogenesis, and anti-proliferative activities against prostate [17,18] and breast cancer cells [19]. Recently, Cokluk et al. [19] observed that RTP cytotoxic and apoptotic effects in hormone-sensitive mammary tumor cells. Moreover, Lin and Yeh [18] found that RTP inhibits the size and weight of prostate tumors in orthotopic TRAMP-C1 tumor-bearing mice, potentially through Th1-polarized immune balancing. Additionally, studies have noted that RTP inhibits angiogenesis through the vascular endothelial growth factor receptor mediated Ak strain transforming/mammalian target of rapamycin/p70S6K signaling pathway and have identified it as a potential drug candidate for cancer prevention and treatment [20]. In addition, ESCC accounts for 90% of all esophageal cancer cases in Asia and Africa [1]. However, there is a scarcity of research on the application of RTP for ESCC treatment, and the mechanisms through which RTP suppresses tumor proliferation remain unclear. For this reason, this study used human esophageal squamous cell carcinoma cell line, CE81T/VGH, to perform in vitro and in vivo studies (a xenograft study in the nude mouse line BALB/cAnN) to investigate the issues mentioned above, with CDDP used in the positive control group.

## 2. Results

### 2.1. Effect of RTP and CDDP on the Growth of Human Esophageal Squamous Cell Carcinoma Cell Line CE81T/VGH

**Figure 1:**
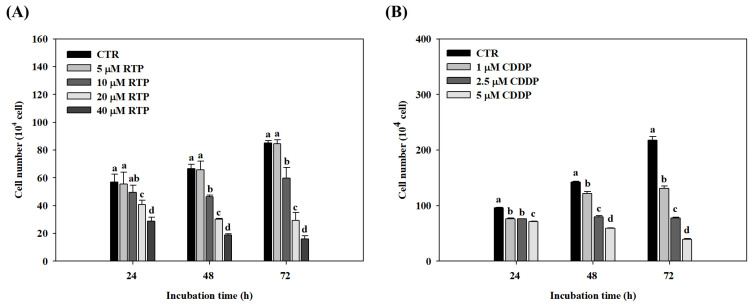
Effects of rutaecarpine (RTP); (**A**) and cisplatin (CDDP);( **B**) on cell growth in human esophageal squamous cells CE81T/VGH. Cells were treated with RTP (5–40 μM) or CDDP (1–5 μM) for 24, 48, and 72 h. CTR represents the control group. Values (means ± SD, *n* = 3) within the same time interval not sharing a common lower case letter are significantly different from one another (*p* < 0.05).

The viability of CE81T/VGH cells was measured using a cell counter following incubation with RTP (5–40 μM) for 24, 48, and 72 h. The viability results are shown in Figure 1A. No significant differences (*p* > 0.05) were observed between the 5 μM RTP treatment group and the control group at any of the time intervals. In contrast, CE81T/VGH cell growth was significantly (*p* < 0.05) inhibited in a dose- and time-dependent manner in the 10, 20, and 40 μM RTP treatment groups, compared to the control group. In the 10, 20, and 40 μM RTP treatment groups, the 24-h inhibitory rate was 13%, 28%, and 49%, respectively; the 48-h inhibitory rate was 31%, 55%, and 74%, respectively; and the 72-h inhibitory rate was 30%, 65%, and 81%, respectively. Furthermore, CE81T/VGH cell growth was significantly (*p* < 0.05) inhibited in the 1, 2.5, and 5 μM CCDP treatment groups in a dose- and time-dependent manner, compared to the control group (see Figure 1B). In the 1, 2.5, and 5 μM CCDP treatment groups, the 24-h inhibitory rate was 20%, 21%, and 26%, respectively; the 48-h inhibitory rate was 14%, 44%, and 59%, respectively; and the 72-h inhibitory rate was 40%, 64%, and 82%, respectively. We, therefore, adopted an RTP dose of 5, 10, and 20 μM in the subsequent experiments, and 5 μM CDDP was used as the positive control.

### 2.2. Effect of RTP on the Cell Cycle of Human Esophageal Squamous Cell Carcinoma Cell Line CE81T/VGH

**Figure 2:**
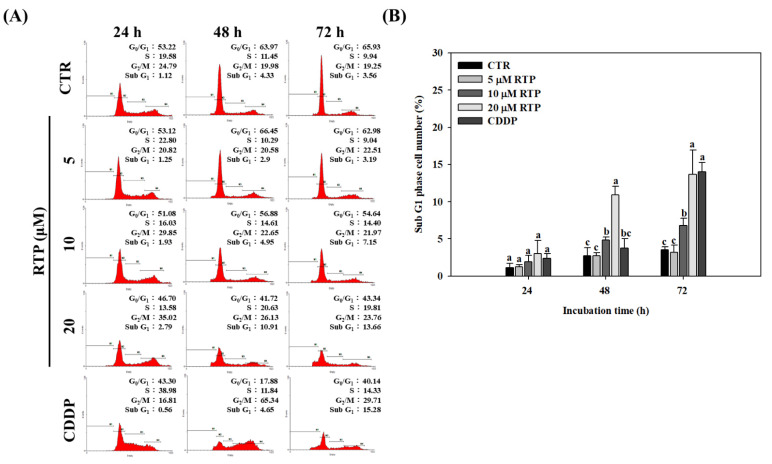
Effects of rutaecarpine (RTP) on cell cycle progression in human esophageal squamous cells CE81T/VGH. Cells (1 × 10^5^ cells/mL) were treated with RTP (5, 10, and 20 μM) or cisplatin (CDDP; 5 μM) for 24, 48, and 72 h. (**A**): Cell cycle distributions were measured using flow cytometry. (**B**): Percentages of sub-G1 cells in Figure 2A. CTR represents the control group. Values (means ± SD, *n =* 3) within the same time interval not sharing a common lower case letter are significantly different from one another (*p* < 0.05).

To determine whether RTP inhibits CE81T/VGH cell growth by altering the cell cycle, we performed further analyses using flow cytometry. As shown in Figure 2A, the flow cytometry results showed that G_2_/M phase cell cycle arrest was induced in a dose-dependent manner in the CE81T/VGH cells following 24-h and 48-h incubation with RTP. The percentage of cells in the 20 µM RTP treatment group increased by 40% and 31% at 24 and 48 h, respectively, compared with the tumor group. Our findings also showed that S phase cell cycle arrest was induced in a dose-dependent manner in the CE81T/VGH cells following 48-h and 72-h incubation with RTP. The percentage of cells in the 20 µM RTP treatment group increased by 80% and 99% at 48 and 72 h, respectively, compared with the control group. Additionally, compared to the control group, the percentage of cells in the sub G_1_ phase increased significantly in a dose- and time-dependent manner after RTP treatment. As shown in Figure 2B, at 72 h, the percentage of cells in the 10 and 20 µM RTP treatment groups increased significantly (*p <* 0.05) by 91% and 283%, respectively, compared to the control group. The percentage of cells in the CDDP treatment group was also significantly higher than the control group by 294%, but this difference was not found to be significant (*p* > 0.05) when compared to the 20 µM RTP treatment group.

### 2.3. Effect of RTP on the Apoptosis of Human Esophageal Squamous Cell Carcinoma Cell Line CE81T/VGH

**Figure 3:**
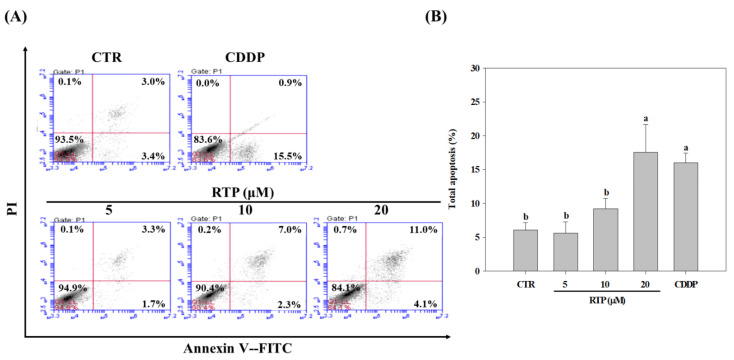
Effects of rutaecarpine (RTP) on apoptosis of human esophageal squamous cells CE81T/VGH at 72 h. Cells (1 × 10^5^ cells/mL) were treated with RTP (5, 10, and 20 μM) or cisplatin (CDDP; 5 μM) for 72 h. (**A**): Annexin V-FITC/PI staining cells were measured using flow cytometry. (**B**): The percentages of apoptosis among different experiment groups. CTR represents the control group. Values (means ± SD, *n =* 3) not sharing a common lower case letter are significantly different (*p* < 0.05).

To understand whether RTP induces apoptosis of CE81T/VGH cells, flow cytometry was again used for further analysis. As shown in Figure 3B, the results showed that apoptosis was induced in the 10 and 20 µM RTP treatment groups in a dose-dependent manner. Compared to the control group, the percentage of cells increased significantly (*p* < 0.05) by 52% and 190% in the 10 and 20 µM RTP treatment groups, respectively. The percentage of cells in the CDDP treatment group was also significantly higher than that of the control group (by 169%), but this difference was not statistically significant (*p* > 0.05) when compared to the 20 µM RTP treatment group.

### 2.4. Effect of RTP on the Expressions of Human Esophageal Squamous Cell Carcinoma Cell Line CE81T/VGH, and p53, Bcl-2, and Bax Proteins

**Figure 4:**
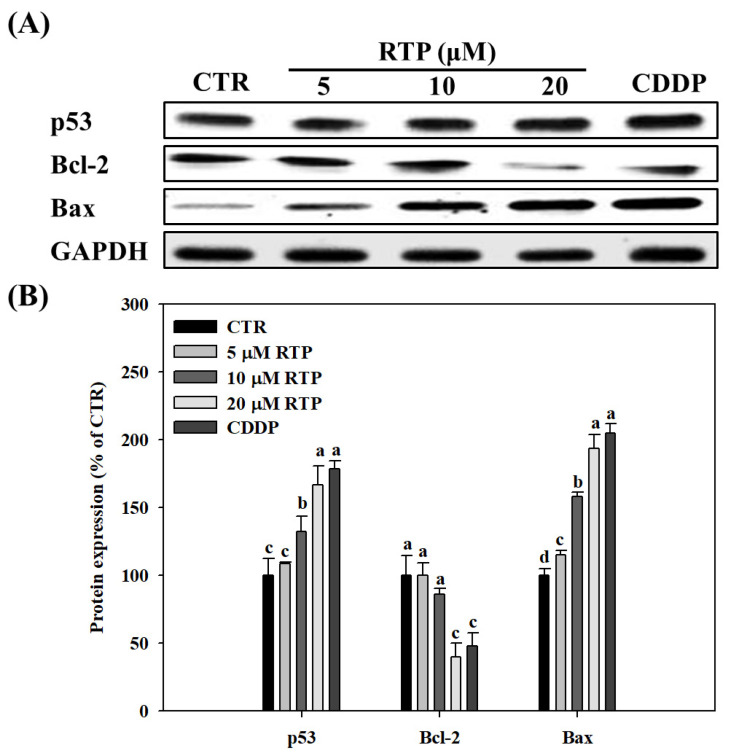
Effects of rutaecarpine (RTP) on expression of p53, Bcl-2, and Bax proteins in human esophageal squamous cells CE81T/VGH. Cells (1 × 10^5^ cells/mL) were treated with RTP (5, 10, and 20 μM) or cisplatin (CDDP; 5 μM) for 72 h. (**A**): Western blots of p53, Bcl-2, and Bax proteins and glyceraldehyde-3-phosphate dehydrogenase (GAPDH). (**B**): Densitometric analysis of Panel A. CTR represents the control group. Values (mean ± SD, *n* = 3) not sharing a common lower case letter in each protein expression are significantly different (*p* < 0.05).

To understand the possible mechanism of action of apoptosis induced by RTP, we analyzed the expression of p53, Bcl-2 B-cell lymphoma 2 (Bcl-2), Bcl-2-associated X (Bax) proteins using Western blot analysis. As shown in Figure 4B, the results showed that p53 and Bax expression increased significantly (*p* < 0.05) in the RTP treatment groups in a dose-dependent manner. Although p53 and Bax expression increased by 69% and 94%, respectively, in the 20 µM RTP treatment group, these increases were not significant (*p* > 0.05) when compared with the CDDP treatment group. Additionally, our findings showed that Bcl-2 protein expression was decreased significantly (*p* < 0.05) in the RTP treatment groups in a dose-dependent manner. Although Bcl-2 expression was decreased by 60% in the 20 µM RTP treatment group, its expression was not significantly different from that of the CDDP treatment group (*p* > 0.05).

### 2.5. Effect of RTP on Tumor Growth in the Nude Mice Xenograft Model 

**Figure 5:**
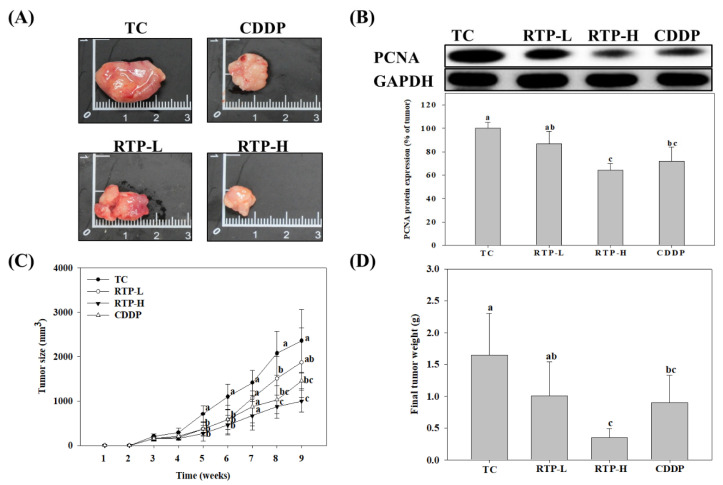
Effects of rutaecarpine (RTP) on (**A**) gross tumor tissue morphology and size; (**B**) protein expression of proliferating cell nuclear antigen (PCNA); (**C**) tumor size; and (**D**) tumor weight in tumor-bearing mice. Male tumor-bearing nude mice were treated with RTP or cisplatin (CDDP) as described in the Methods. The tumor control (TC) was administered the vehicle only. TC: tumor only; RTP-L: RTP by oral gavage at 25 mg/kg; RTP-H: RTP by oral gavage at 75 mg/kg; CDDP: intraperitoneally injected CDDP at 5 mg/kg. Values (means ± SD, *n =* 9) not sharing a common lower case letter are significantly different (*p* < 0.05).

To determine whether the results of the cell models were consistent with in vivo experimental findings, we further investigated the effects of RTP on tumor growth in the nude mice xenograft model. As shown in Figure 5C, the results showed that, with the exception of the RTP-L (25 mg/kg) treatment group, tumor size was significantly (*p* < 0.05) inhibited in the RTP-H (75 mg/kg) and CDDP (5 mg/kg) treatment groups. Similar findings were also noted in the tumor weight measurements. As shown in Figure 5D, compared to the tumor group, the inhibitory rate in the RTP-H and CDDP treatment groups was 79% and 45%, respectively. Additionally, we also examined the expression of proliferating cell nuclear antigen (PCNA) protein in tumor tissue. As shown in Figure 5B, PCNA protein expression was significantly (*p* < 0.05) inhibited at a rate of 32% in the RTP-H treatment group compared to the tumor group, while the inhibitory effects were not significant (*p* > 0.05) in the RTP-L treatment group. PCNA protein expression was also significantly reduced in the CDDP treatment group compared to the tumor group, but this reduction was not significant (*p* > 0.05) when compared with the RTP-H treatment group.

### 2.6. Effect of RTP on p53, Bcl-2, Bax, Caspase-9, and Caspase-3 Protein Expression in Tumor Tissue in the Nude Mice Xenograft Model 

**Figure 6:**
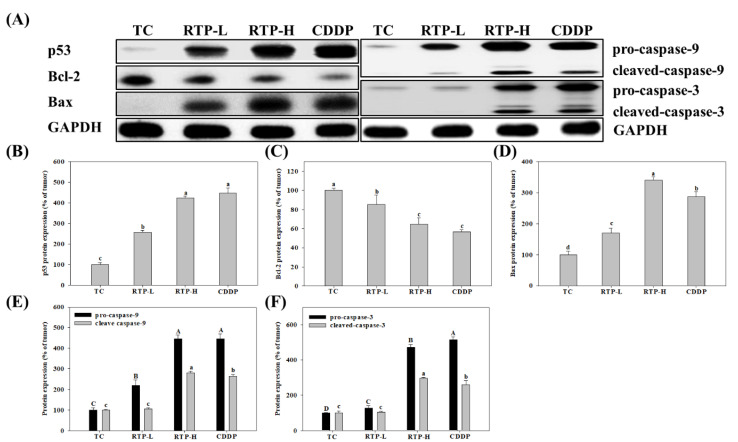
Effects of rutaecarpine (RTP) on (**B**) p53; (**C**) Bcl-2; (**D**) Bax; (**E**) caspase-9; and (**F**) caspase-3 protein expression in tumor tissue from tumor-bearing mice. Male tumor-bearing nude mice were treated with RTP or cisplatin (CDDP) as described in the Methods. The tumor control (TC) was administered the vehicle only. (**A**): Western blots of protein and glyceraldehyde-3-phosphate dehydrogenase (GAPDH) (**B**–**F**): Densitometric analysis of Panel A. Values (mean ± SD, *n* = 9) not sharing a common capital (or a lower case letter) in each protein expression are significantly different (*p* < 0.05). TC: tumor only; RTP-L: RTP by oral gavage at 25 mg/kg; RTP-H: RTP by oral gavage at 75 mg/kg; CDDP: intraperitoneally injected CDDP at 5 mg/kg.

To elucidate the possible mechanisms of tumor growth inhibition by RTP, we analyzed the expression of p53, Bcl-2, Bax, caspase-9, and caspase-3 proteins in tumor tissue. As shown in Figure 6, the results showed that p53 protein expression significantly (*p* < 0.05) increased by 157% and 324% in the RTP-L and RTP-H treatment groups, respectively, compared with the tumor group. In the CDDP treatment group, p53 protein expression increased significantly (*p* < 0.05) by 347% compared with the tumor group, but this increase was not significant (*p* > 0.05) when compared with the RTP-H treatment group. Furthermore, we found that Bcl-2 protein expression significantly decreased by 15% and 35% in the RTP-L and RTP-H treatment groups, respectively, compared with the tumor group (see Figure 6C). In the CDDP treatment group, Bcl-2 protein expression significantly (*p* < 0.05) decreased by 43% compared with the tumor group, but this increase was not significant (*p* > 0.05) when compared with the RTP-H treatment group. Additionally, the expression of the pro-apoptotic protein Bax significantly (*p* < 0.05) increased by 70% and 241% in the RTP-L and RTP-H treatment groups, respectively, compared with the tumor group. Bax expression also significantly (*p* < 0.05) increased by 187% in the CDDP treatment group compared with the tumor group, but this increase was significantly (*p* > 0.05) lower than that observed in the RTP-H treatment group. The expression of the caspase family proteases is essential for the induction of apoptosis. Our results showed that RTP significantly increases the expression of cleaved caspase-9 and cleaved caspase-3 proteins by 181% and 197%, respectively, in the RTP-H treatment group compared to the tumor group. The expression of cleaved caspase-9 and cleaved caspase-3 proteins increased by 165% and 161%, respectively (*p* < 0.05), in the CDDP treatment group compared to the tumor group, but these increases were significantly (*p* < 0.05) lower than those observed in the RTP-H treatment group.

### 2.7. Effect of RTP Toxicity in the Nude Mice Xenograft Model 

**Figure 7:**
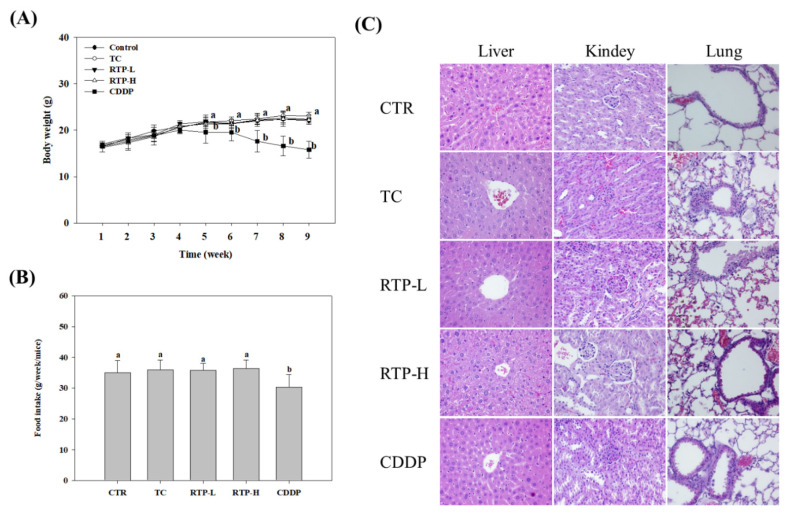
The (**A**) body weight, (**B**) food intake, and (**C**) hematoxylin and eosin staining of major organs in tumor-bearing mice. Male tumor-bearing nude mice were treated with rutaecarpine (RTP) or cisplatin (CDDP) as described in the Methods. The control (CTR) and tumor control (TC) were administered the vehicle only. Values (means ± SD, *n =* 9) not sharing a common lower case letter are significantly different (*p* < 0.05). TC: tumor only; RTP-L: RTP by oral gavage at 25 mg/kg; RTP-H: RTP by oral gavage at 75 mg/kg; CDDP: intraperitoneally injected CDDP at 5 mg/kg.

As shown in Figure 7A,B, no significant differences (*p* > 0.05) were observed in the body weight and food intake of nude mice in the tumor, RTP-L, and RTP-H treatment groups, compared with the control group. However, a significant (*p* > 0.05) reduction in body weight and food intake was observed in the CDDP treatment group from the fifth week onwards. Pathological tissue biopsies of the liver, kidney, and lung revealed no pathological changes in any treatment group compared to the control group (see Figure 7C). Several studies have shown that CDDP can cause nephrotoxicity by reducing glomerular filtration and increasing blood urea nitrogen (BUN) and creatinine (Cre) levels in the blood [21,22,23]. As shown in Table 1, compared with the control group, no significant differences (*p* > 0.05) were observed in the serum BUN and Cre levels in the RTP-L and RTP-H treatment groups. However, in the CDDP treatment group, serum levels of BUN and Cre increased significantly (*p* < 0.05) by 24% and 33%, respectively. Additionally, hematological analysis revealed that no significant differences (*p* > 0.05) were observed in the red blood cells (RBC) count, hemoglobin (Hb) level, hematocrit (HCT) level, mean corpuscular hemoglobin (MCH), and mean corpuscular hemoglobin concentration (MCHC) of the RTP-L and RTP-H treatment groups, compared with the control group. Similar findings were also observed in the CDDP treatment group, though the MCHC was lower than the other groups.

## 3. Discussion

The mortality rate of esophageal cancer is relatively high worldwide, and, currently, no effective treatment strategies are available for the cancer [1]. The results of this study suggest that RTP can inhibit the growth of the human esophageal squamous cell carcinoma cell line CE81T/VGH in vivo and in vitro, and induce apoptosis. Cell assays revealed that RTP significantly inhibits CE81T/VGH growth in a dose- and time-dependent manner, and results from xenograft modeling indicated a significant (*p* < 0.05) reduction in tumor size and weight in the high-dose RTP treatment group. A study by Fridman et al. [24] demonstrated that PCNA (an important indicator of tumor proliferation [25] can inhibit the cyclin-dependent p21 protein, as both compete to bind with DNA polymerase δ. Increased PCNA expression, thus, promotes tumor cell proliferation [26]. In our study, RTP was found to significantly (*p* < 0.05) reduce PCNA expression in tumor tissue, potentially by inhibiting tumor size and weight. This finding was consistent with the results of our cell assay.

There is lack of studies in the scientific literature pertaining to RTP-induced tumor cell apoptosis. One study examined the cytotoxicity of rutaecarpine analogues in vitro *and* noted that RTP inhibits the proliferation of human lung cancer A549, rectal cancer HT-29, and ovarian cancer OVCAR-4 cell lines [27]. Another study determined that RTP promotes the cytotoxic and apoptotic effects of hormone-sensitive breast cancer cells [19]. We analyzed the cell cycle and apoptotic processes and found that RTP induces arrest of CE8T/VEGH cells in the G_2_/M and S phases, and significantly (*p* < 0.05) increases distribution of cells in the Sub G_1_ phase. Cell death can occur through programed apoptosis and non-programed necrosis [28]. To determine whether cell death was occurring through the former, we used flow cytometry to perform apoptosis assays, which showed that RTP induces CE81T/VGH apoptosis, particularly following an incubation time of 72 h. These results are the first to suggest that RTP has the potential to induce apoptosis of human esophageal squamous cell carcinoma cells.

When DNA is damaged, activation of the p53 gene promotes p21 protein expression and inhibits the cyclin and cyclin-dependent kinase-1 (Cdk1) proteins, resulting in G_2_/M phase arrest and promoting DNA repair [29,30,31]. However, when severe DNA damage occurs, the p53 gene instead triggers signals for apoptosis, and induces apoptosis of proteins such as Bcl-2 and Bax [29,30,32,33]. Yin et al. [34] found that diallyl disulfide induces apoptosis and G_2_/M phase arrest of the human esophageal squamous cell carcinoma cell line ECA109. This mechanism is associated in part with caspase-3 activation and Bax/Bcl-2 ratio upregulation. Furthermore, Wang et al. [32] reported that matrine, a bioalkaloid present in plants, upregulates p53 and p21 protein expression and downregulates Bcl-2 protein expression in vitro, thus inducing apoptosis in the ECA109 cell line. In this study, the results of the cell assay revealed that RTP significantly (*p* < 0.05) enhances p53 protein expression, inhibits Bcl-2 protein expression, and further enhances Bax protein expression in a dose-dependent manner (*p* < 0.05). The results of the xenograft model and our analysis of protein expression in tumor tissue further support these findings.

The caspase family of proteases participate in irreversible apoptotic reactions, in which cytochrome C released from the mitochondria binds with Apaf-1 and inactivated procaspase-9 to form the apoptosome. The activated procaspase-9 is then cleaved into cleaved caspase-9, which subsequent catalyzes an apoptotic cascade that cleaves procaspase-3 into cleaved caspase-3, thus activating it [33,35,36]. For instance, a study by Zou et al. [33] reported that gimatecan induces apoptosis in esophageal squamous cell carcinoma cell lines in vitro by increasing Bax expression, activating cleaved caspase-3 and cleaved-caspase-9, and reducing Bcl-2 expression. This study further analyzed the expression of caspase-9 and caspase-3 proteins in tumor tissue. The results revealed that expression levels of inactivated proteins (procaspase-9 and procaspase-3) and activated increased cleaved proteins (caspase-9 and caspase-3) were significantly (*p* < 0.05) higher in the high-dose RTP treatment group. We, therefore, postulate that RTP initially induces G_2_/M phase cell cycle arrest in CE81T/VGI cells (*p* < 0.05). As the severity of DNA damage increases with incubation time, the p53 gene is activated, followed by inhibition of Bcl-2 expression and increased Bax expression. This results in increase protein expression of cleaved caspase-9 and cleaved caspase-3, and ultimately induces CE81T/VGH cell apoptosis. 

Yang et al. [37] intravenously injected RTP into the tail veins of Kunming mice and reported that the LD_50_ of RTP was 65 mg/kg. In our animal experiments, RTP was tube-fed to the nude mice at a dose of 75 mg/kg in the RTP-H group. This method of administration allowed us to investigate whether RTP had any physiological toxicity in the mice. Based on the body weight and food intake of the mice, no significant differences (*p* > 0.05) were observed between the RTP treatment groups and the control group, though the CDDP treatment group showed a significant decrease. No significant differences (*p* > 0.05) were observed in the hematology test results or serum BUN and Cre measurements across any of the RTP treatment groups compared to the control group. However, the serum BUN and Cre levels increased significantly (*p* < 0.05) in the CDDP treatment group. Severe side effects, including nephrotoxicity and neurotoxicity, are concomitant with CDDP use [20,21]. Previous studies have shown that CDDP therapy can cause nephrotoxicity by reducing glomerular filtration and increasing blood BUN and Cre levels [21,22,23], and this is supported by our findings. Furthermore, our pathological biopsies indicated that no pathological changes to the liver, kidneys, lungs, or other major organs were observed in the RTP treatment groups. This suggests that the RTP dose and administration routes used in this study did not result in genotoxicity or hematotoxicity.

## 4. Materials and Methods

### 4.1. Reagents

All chemicals used were of reagent grade or higher. Dulbecco’s Modified Eagle Medium (DMEM), fetal bovine serum, trypsin, penicillin, streptomycin, sodium pyruvate, and nonessential amino acids were sourced from GIBCO/BRL (Rockville, MD, USA). Rutaecarpine (purity: >98%; 8,13-Dihydroindolo [2′,3′:3,4] pyrido [2,1-b] quinazolin-5 (7H)-one) and CDDP were sourced from Merck KGaA (Darmstadt, Germany), while DMSO was sourced from Tedia Co. (Fairfield, OH, USA). Anti-p53 and glyceraldehyde-3-phosphate dehydrogenase (GAPDH). mouse monoclonal antibody, and anti-Mouse IgG antibody were purchased from GeneTex, Inc. (Irvine, CA, USA). Mouse monoclonal antibodies specific for Bax, Bcl-2, caspase-3, caspase-9, and PCNA were purchased from Santa Cruz Biotechnology (Dallas, TX, USA).

### 4.2. Animal Study

The protocol for this study was approved by the Animal Research Committee of Hungkuang University (IACUC approval no. 10403). Male nude mice aged five to six weeks were obtained from the National Laboratory Animal Center (Taipei, Taiwan). The animals were housed individually in hanging wire mesh cages with controlled temperature (23 ± 2 °C) and humidity (65 ± 5%), and an alternating 12 h light/dark cycle. Upon arrival, animals were acclimated for one week, during which they were fed a standard rodent diet (Lab 5001, Purina Mills, St. Louis, MO) and water *ad libitum*. Following this, the animals were subcutaneously injected with CE81T/VGH cells at a dose of 2.5 × 10^6^ cells (in 200 μL of matrigel; BD Biosciences, Franklin Lakes, NJ, USA) in the right flank. Tumor nodule volumes were measured once a week using the following formula: (L1 × L22)/2, where L1 is the long axis and L2 is the short axis of the tumor [38]. Two weeks following cell injection, tumor nodules were palpable. The animals were then randomly assigned to the following five groups (*n* = 9 per group) for an additional six weeks: control (CTR), tumor control (TC; tumor only), RTP-low dose (RTP-L), RTP-high dose (RTP-H), and CDDP. RTP was administered twice weekly (RTP-L and RTP-H, 25 and 75 mg/kg body weight melted in carboxymethyl cellulose solution, respectively) by oral gavage, while CDDP was administered twice weekly (5 mg/kg body weigh melted in a physiologic solution) by intraperitoneal injection. The mice in the CTR and TC group were treated with drug vehicles. The doses of RTP were designed according to the study by Yang et al. [37] and Jeon et al. [39]. All animals were allowed free access to a standard rodent diet (Lab 5001, Purina Mills, St Louis, MO, USA) and water during the study. The body weights of the mice were recorded weekly. Blood samples were collected from the retro-orbital plexus of the nude mice under deep isoflurane anesthesia at nine weeks to determine serum BUN and creatinine, RBC, MCH, MCHC, Hb, and HCT levels. At the end of the experiment, all animals were euthanized using CO_2_ asphyxiation. The tumor, liver, lungs, and kidneys were collected and stored at −80 °C until analysis. In addition, samples from the liver, lungs, and kidneys were also resected, fixed, and sectioned for H&E (hematoxylin and eosin) staining to determine any organ-specific toxicity.

### 4.3. Cell Culture

Human esophageal squamous cells CE81T/VGH were obtained from the Bioresource Collection and Research Center (Hsinchu, Taiwan) and were cultured in DMED 10% (*v*/*v*) fetal bovine serum, 0.37% (*w*/*v*) NaHCO_3_, penicillin (100 units/mL), and streptomycin (100 μg/mL) at 37 °C in a humidified incubator with 5% CO_2_ and 95% air. An equal number (1 × 10^5^ cells/mL) of cells were incubated for 24 h prior to the various treatments. Before the experiment, the medium was removed, and the cells were washed twice with PBS. New media (with 10% fetal bovine serum) containing 5–40 μM RTP or 1–5 μM CDDP was then added, and the samples were incubated for 24, 48, and 72 h. RTP and CDDP were freshly prepared as 20 mM or 4 mM stock solutions, which were then dissolved in DMSO or 0.9% normal saline. Before use, the compounds were diluted in 10% fetal bovine serum in culture medium to the desired concentrations at the time of addition. The highest concentration of DMSO used did not exceed 0.1% (v:v) of the total assay volume, and, therefore, did not affect cell viability.

### 4.4. Cell Growth Analysis

CE81T/VGH cells were plated in 6-well plates at a density of 1×10^5^ cells/well and grown for 24, 48, and 72 h. Different concentrations of RTP or CDDP were then added to the cells to reach final concentrations of 5–40 μM or 1–5 μM, respectively, in the presence of FBS. Control groups contained 10% FBS only. The cells were then grown at 37 °C, with 5% CO_2_ and 95% air for different periods of time. Trypan blue exclusion protocol was subsequently used to determine cell viability.

### 4.5. Cell Cycle Analysis

The effect of RTP on the cell cycle distribution of CE81T/VGH was determined using flow cytometry as previously described [40]. Briefly, approximately 1 × 10^5^ cells/well of CE81T/VGH cells were incubated in six-well plates with RTP (5–20 μM) or CDDP (5 μM) for different time periods, after which the cells were harvested by centrifugation (100 × *g*, 3 min). The cells were collected and fixed with ice-cold 70% ethanol (in PBS) overnight at 4 °C. Following centrifugation, the cell pellets were resuspended in PBS containing 4 μg/mL propidium iodide, 0.5 mg/mL RNase (Sigma-Aldrich, St. Louis, MO, USA), and 1% Triton X-100 for 30 min at 37 °C. Subsequently, the cells were analyzed in a FACScalibur system (BD Biosciences, San Jose, CA, USA) using CellQuest software (Becton Dickinson, Franklin Lakes, NJ, USA). The percentage of cell cycle phases was analyzed using WinMDI 2.8 software.

### 4.6. Annexin V-FITC-Propidium Iodide Assay

An Annexin V-FITC apoptosis detection kit (BD Pharmingen, San Diego, CA, USA) was used to determine the number of apoptotic cells as described in our previous study [41]. According to the manufacturer’s instructions, the treated cells were harvested after the indicated time, washed twice with ice-cold PBS, and resuspended in 100 μL binding buffer. An aqueous solution of Annexin V-FITC and propidium iodide staining buffer was then added, and the mixture was incubated in darkness at 37 °C for 15 min. Before flow cytometric analysis, 400 μL of binding buffer was added to each sample. A total of 100,000 events per sample were analyzed. Flow cytometric analysis was performed with a FACS Calibur flow cytometer (BD Biosciences, Franklin Lakes, NJ, USA) using WinMDI 2.8 software.

### 4.7. Western Blotting

Expression levels of p53, Bcl-2, Bax, caspase-9, caspase-3, and PCNA proteins were determined using Western blotting as previously described [40]. Briefly, the medium was removed, and cells were lysed with 20% SDS containing 1 mM PMSF. The lysate was sonicated for 30 s on ice, followed by centrifugation at 12,000 × *g* for 30 min at 4 °C. 40 μg of protein from the supernatant was resolved by sodium dodecyl sulfate polyacrylamide gel electrophoresis and transferred onto a nitrocellulose membrane. After blocking with Tris-buffered saline buffer (20 mM Tris-HCl, 150 mM NaCl, pH 7.4) containing 5% nonfat milk, the membrane was incubated with anti-p53, Bcl-2, Bax, caspase-9, caspase-3, and PCNA monoclonal antibody, followed by horseradish peroxidase-conjugated anti-mouse IgG, then visualized using an ECL chemiluminescent detection kit (Amersham, Buckinghamshire, UK). The relative levels of p53, Bcl-2, Bax, caspase-9, caspase-3, and PCNA proteins were quantitated using Matrox Inspector version 2.1 (Matrox Electronic Systems Ltd., Dorval, Canada) software.

### 4.8. Hematological Assay

The blood samples were analyzed for hematological assay using an automated hematology analyzer (Sysmex, Kobe, Japan) to determine RBC, MCH, MCHC, Hb, and HCT levels.

### 4.9. Biochemical Assay

The serum BUN and creatinine concentrations were measured using appropriate commercial assay kits according to the manufacturer’s protocol (Randox laboratories Ltd., Antrim, UK; kits cat. No. UR 221 and CR510, respectively).

### 4.10. Histopathological Examination

A portion of fresh liver, lung, or kidney tissue was fixed using 10% formalin, then placed in an embedding box. After dehydration overnight, this piece was embedded with paraffin at −20 °C and cut into 3 μm sections. Finally, the tissue sections were stained with H&E, followed by microscopic examination.

### 4.11. Statistical Analysis

Values are expressed as means ± SDs and were analyzed using one-way analysis of variance followed by Duncan’s multiple range test for comparison of group means. Differences are considered statistically significant at *p* < 0.05.

## 5. Conclusions

The results of our experiments suggest that RTP may lead to increased p53 protein expression in vivo and in vitro, and thereby increase Bax protein expression, inhibit Bc1-2 protein expression, and increase caspase-9 and caspase-3 downstream, thus promoting apoptosis of esophageal squamous cell carcinoma cells and inhibiting tumor growth. Furthermore, to the best of our knowledge, this is the first study to report the potential of RTP for inducing apoptosis in human esophageal squamous cell carcinoma cells, which supports its clinical use as a potent anticancer drug.

## Figures and Tables

**Table 1:** Effects of rutaecarpine (RTP) on hematological parameters, blood urea nitrogen (BUN), and creatinine (Cre) of tumor-bearing mice ^1^.

Group	RBC (×10^6^/µL)	Hb (g/dL)	HCT (%)	MCH (Pg)	MCHC (%)	BUN (mg/dL)	Cre (mg/dL)
CTR ^2^	6.68 ± 0.6 ^a,3^	11.5 ± 0.7 ^a^	32.6 ± 2.6 ^a^	17.2 ± 0.4 ^a^	35.2 ± 0.8 ^a3^	32.9 ± 2.7 ^c^	0.337 ± 0.012 ^b^
TC	7.26 ± 0.2 ^a^	12.1 ± 0.4 ^a^	34.7 ± 0.7 ^a^	16.6 ± 0.4 ^a^	34.8 ± 1.2 ^a^	36.3 ± 0.5 ^b^	0.345 ± 0.005 ^b^
RTP-L	6.34 ± 0.9 ^a^	12.0 ± 0.8 ^a^	32.4 ± 0.6 ^a^	17.9 ± 1.4 ^a^	35.6 ± 0.5 ^a^	34.2 ± 1.3b ^c^	0.333 ± 0.006 ^b^
RTP-H	7.46 ± 0.6 ^a^	12.1 ± 0.4 ^a^	33.3 ± 2.0 ^a^	18.1 ± 0.5 ^a^	36.0 ± 1.0 ^a^	34.9 ± 0.9b ^c^	0.333 ± 0.012 ^b^
CDDP	6.65 ± 0.4 ^a^	11.2 ± 0.4 ^a^	33.9 ± 2.9 ^a^	16.9 ± 0.4 ^a^	34.1 ± 1.3 ^b^	40.7 ± 2.0 ^a^	0.447 ± 0.006 ^a^

^1^ Male tumor-bearing nude mice were treated with rutaecarpine (RTP) or cisplatin (CDDP) as described in the Methods.^2^ The control (CTR) and tumor control (TC) group were administered the vehicle only. TC: tumor only; RTP-L: RTP by oral gavage at 25 mg/kg; RTP-H: RTP by oral gavage at 75 mg/kg; CDDP: intraperitoneally injected CDDP at 5 mg/kg. ^3^ Values (means ± SD, *n* = 9) not sharing a common lower case letter are significantly different (*p* < 0.05) among groups in the same item.

## Data Availability

The data presented in this study are available in the published article.
